# Network Analysis of Social Anxiety, Avoidant Behavior, and Spiritual Well‐Being in Breast Cancer Patients: A Pathway to Targeted Psychosocial Interventions

**DOI:** 10.1155/da/9076098

**Published:** 2026-08-07

**Authors:** Cheng Yang, Jiawei Li, Yang Wen, Peijin He, Qin Zeng

**Affiliations:** ^1^ Department of Pediatric Intensive Care Unit Nursing, West China Second University Hospital, Sichuan University, Chengdu, Sichuan, China, scu.edu.cn; ^2^ Department of Pediatric Intensive Care Unit Nursing, WCSUH-Tianfu·Sichuan Provincial Children’s Hospital, Meishan, Sichuan, China; ^3^ Key Laboratory of Birth Defects and Related Diseases of Women and Children, Sichuan University, Ministry of Education, Chengdu, Sichuan, China, moe.edu.cn; ^4^ Department of Pediatric Outpatient Nursing, West China Second University Hospital, Sichuan University, Chengdu, Sichuan, China, scu.edu.cn; ^5^ Department of Pediatric Gastroenterology Nursing, West China Second University Hospital, Sichuan University, Chengdu, Sichuan, China, scu.edu.cn

**Keywords:** avoidance behavior, breast cancer, oncology nursing, psychological adaptation, psychosocial care, social anxiety, spiritual health, symptom network

## Abstract

**Background:**

Social anxiety and avoidance behaviors are prevalent among breast cancer patients, yet their interrelationships with protective factors such as spiritual well‐being remain poorly understood. Network analysis offers a novel approach to identify central and bridge nodes that may serve as key targets for psychosocial intervention.

**Methods:**

We conducted a cross‐sectional study of 302 female breast cancer patients from a tertiary hospital in China. Participants completed the Social Anxiety Scale (SAS_1), Social Avoidance Scale (SAS_2), and the Functional Assessment of Chronic Illness Therapy–Spiritual Well‐Being (FACIT‐Sp). A network model was constructed to examine the interrelationships among these constructs, with centrality and bridge centrality indices used to identify influential nodes.

**Results:**

Spiritual well‐being—particularly items such as “strength from faith” and “confidence in recovery”—showed the highest strength centrality, indicating its central role in the psychological adaptation network. Social avoidance emerged as the strongest bridge node, connecting spiritual well‐being with social anxiety. Surgical approach did not significantly alter the global network structure, but subgroup analyses revealed distinct node‐level bridge strengths, suggesting differential psychological adaptation pathways.

**Conclusion:**

Spiritual well‐being occupies a central position in the psychological network of breast cancer patients, and social avoidance shows the strongest bridging function between spiritual resources and emotional symptoms. These findings support the integration of spiritual assessment and targeted avoidance‐reduction strategies into supportive care. Future research should validate these networks in larger samples and develop node‐specific interventions to improve psychological adjustment.

## 1. Introduction

Breast cancer is the most commonly diagnosed malignancy among women worldwide and represents a significant public health concern [1, 2]. According to a World Health Organization (WHO) report, breast cancer incidence shows a “worrisome rising trend.” Projections indicate that without effective interventions, global breast cancer incidence and mortality rates will increase by 40.8% and 51.9%, respectively, by 2040 [3]. In the United States, breast cancer accounts for 32% of all new cancer cases in women, ranking as the most prevalent cancer type [4]. With advancements in screening and treatment, the 5‐year relative survival rate has improved from 49% in the 1970s to 69% currently [2, 5]. Concurrently, breast health issues are becoming increasingly prominent in China. The incidence and mortality rates of breast cancer in China are increasing at a significantly faster pace compared to those in developed countries such as the United Kingdom and the United States. Breast cancer has become the most common malignant tumor among Chinese women, with its mortality rate also exceeding the global average. Projected data indicate that by 2030, the annual number of new cases in China will reach 452,000, with 98,800 deaths [6]. Against this backdrop, addressing post‐treatment mental health and social adaptation in breast cancer patients carries substantial practical significance [7, 8]. Breast cancer patients often face multiple challenges including altered body image, functional limitations, and social role transitions, which predispose them to stigma, social anxiety, and avoidance behaviors [9]. Recent studies on Chinese survivors demonstrate significantly elevated social anxiety levels, where social comparison tendencies exacerbate anxiety through body image mediation, while self‐concept clarity exerts buffering effects. This suggests that negative body cognition and identity confusion constitute important psychological mechanisms underlying social avoidance [10].

The spiritual health has gained increasing attention as a protective factor for psychological adaptation in cancer patients [11]. It encompasses not only religious beliefs but also perceptions of life meaning, inner peace, and faith strength [12]. Research indicates positive correlations between spiritual health and psychological resilience, with high self‐transcendence (incorporating spiritual dimensions) reducing the disease progression risk by 73% [13]. These findings suggest that spiritual resources may alleviate emotional‐behavioral problems by enhancing psychological adjustment capacity [14].

Although potential associations exist among spiritual health, social anxiety, and avoidance behaviors, their underlying mechanisms remain unclear. A large‑scale study of 1127 breast cancer patients found that avoidant coping (attempts to avoid cancer‑related thoughts and feelings) mediated 19%–54% of the relationship between well‑being domains and distress, suggesting that avoidance behaviors play a pivotal role in psychological adaptation following cancer treatment [15]. Traditional statistical methods struggle to reveal complex interactive relationships between variables, whereas network analysis conceptualizes psychological constructs as dynamic systems that visually display partial correlations and centrality, facilitating identification of key intervention targets [16].

Building on this foundation, the current study proposes to systematically examine the intrinsic relationships between social anxiety, avoidance behaviors, and spiritual health in breast cancer patients using network analysis, aiming to identify central nodes and provide evidence for developing integrated interventions combining spiritual‐psychological support with health education [17].

## 2. Research Methods

### 2.1. Study Design

This study employed a cross‐sectional design.

### 2.2. Study Participants

#### 2.2.1. Sampling Method

This study consecutively enrolled eligible breast cancer patients treated at a tertiary Grade A hospital in Sichuan Province from May to December 2023. To enhance sample representativeness and account for potential impacts of chemotherapy cycles on mood, behavior, and cognition [18], the study employed multi‐timepoint sampling for comprehensive data acquisition. The specific data collection protocol was designed as follows: for patients receiving 4, 6, or 8 total chemotherapy cycles, data were randomly collected during one session from cycles 1–4, cycles 5–6, and cycles 7–8, respectively; for other chemotherapy regimens, data collection occurred during one randomly selected session from the final 1–2 cycles.

#### 2.2.2. Diagnostic and Inclusion/Exclusion Criteria

Diagnostic criteria: Patients pathologically diagnosed with stage I, II, or III breast cancer were included according to the 8th edition Tumor–Node–Metastasis (TNM) staging system of the American Joint Committee on Cancer (AJCC) [19, 20].

Inclusion criteria: (1) Female patients diagnosed with breast cancer confirmed by pathological examination, (2) aged 18 years or older, (3) patients who had completed surgery and at least one cycle of chemotherapy, (4) voluntary participation in the study, and (5) patients with clear consciousness and normal speech.

Exclusion criteria: (1) Patients with cancer recurrence, (2) history or current diagnosis of other malignant tumors, and (3) comorbidities with other major diseases.

Elimination criteria: (1) Questionnaires with >20% missing data and (2) self‐contradictory questionnaires.

#### 2.2.3. Sample Size Estimation

The sample size of this study was estimated based on the formula for cross‐sectional survey sample size calculation. The calculation formula was as follows: *N* = (*Uα* × *σ*/*δ*)^2^. With reference to preliminary survey results and previous literature [21], the standard deviation (*σ*) of the total quality of life scale score was estimated to be 14, while the margin of error (*δ*) was set at 2. After calculation using the formula and considering a 20% dropout rate, the final required sample size was determined to be 226 cases.

In addition, the adequacy of the final sample (*N* = 302) for network analysis was evaluated against the methodological recommendations. Simulation studies have shown that sample sizes of 250–350 are generally sufficient to achieve stable network estimates when the network contains up to 20 nodes and exhibits moderate sparsity [22]. Our network comprised 14 nodes, and the parameter‐to‐sample ratio was approximately 0.30 (91 estimated edges for 302 participants), falling within acceptable ranges.

### 2.3. Research Instruments

#### 2.3.1. General Information Questionnaire

The questionnaire was developed by the researchers and comprised 18 items covering general demographic characteristics (e.g., age, ethnicity, and occupation) and disease‐ and treatment‐related information (e.g., disease duration, tumor stage, and surgical method).

#### 2.3.2. Social Anxiety Scale(SAS_1)

The SAS_1 was adapted from the Self‐Consciousness Scale (SCS) developed by Fenigstein et al. [23] and is used to assess the level of anxiety individuals experience in social situations due to being observed or evaluated by others. The scale consists of six items that capture feelings of tension in contexts such as being in public or interacting with strangers, as well as excessive concern about others’ perceptions. Responses were scored on a 5‐point Likert scale ranging from 0 to 4, with one reverse‐scored item included. Total scores range from 0 to 20, and higher scores indicate greater social anxiety and a stronger fear of negative evaluation. In this study, the scale showed good internal consistency, with Cronbach’s *α* coefficients of 0.817 in the pilot survey and 0.832 in the main survey.

#### 2.3.3. Social Avoidance Scale(SAS_2)

The SAS_2 was derived from the Social Avoidance and Distress Scale (SADS) developed by Watson and Friend [24], with the Chinese version revised by Ma Hong et al. [24, 25]. While the original scale used a “yes/no” dichotomous scoring system, the present study employed a 5‐point Likert scale (ranging from 0 to 4) to enhance measurement sensitivity. Seven items were reverse‐scored. The scale consists of 14 items, with total scores ranging from 0 to 56, with higher scores indicate a stronger tendency toward social avoidance. Cronbach’s *α* coefficients were 0.76 in the pilot survey and 0.82 in the main survey, both exceeding the acceptable threshold of 0.70 and indicating satisfactory internal consistency. The 5‐point version has also shown good reliability (Cronbach’s *α* = 0.85) in Chinese samples [26].

#### 2.3.4. Functional Assessment of Chronic Illness Therapy –Spiritual Well‐Being (FACIT‐Sp)

The Chinese version of the FACIT‐Sp was translated and adapted by Xiangyu et al. [27], comprising three dimensions: peace, meaning, and faith, with a total of 12 items. It employed a 5‐point Likert scale (ranging from 0 to 4), including one reverse‐scored item. The total score ranges from 0 to 48, with scores categorized into three levels: low (<24), moderate (24–35), and high (>36). The original scale demonstrated a content validity of 0.90 and a Cronbach’s *α* coefficient of 0.83. In the present study, the scale exhibited good internal consistency, with Cronbach’s *α* coefficients of 0.887 in the pilot survey and 0.90 in the formal survey.

### 2.4. Data Collection and Quality Control

#### 2.4.1. Data Collection

A pilot test was conducted with 25 patients to refine ambiguous item wordings based on their feedback. The formal survey was then administered anonymously in the oncology ward of a tertiary hospital in Southwest China. Questionnaires were distributed both online (via Questionnaire Star) and on paper. Following departmental approval, researchers explained the study’s purpose and procedures to patients, whose voluntary completion and submission of the questionnaire constituted informed consent. Participants could choose their preferred format. Neutral explanations were provided for any items that were difficult to understand, with family assistance permitted if needed, while ensuring that responses reflected the patients’ true perspectives. Questionnaires were collected immediately upon completion and checked for missing items, which were addressed on the spot to ensure data completeness.

#### 2.4.2. Quality Control

During the study design phase, a scientific and rigorous research protocol was developed through a comprehensive literature review and consultations with experts in oncology and statistics. During the data collection phase, following approval from the hospital’s oncology department, the researchers uniformly distributed and collected the questionnaires. Adherence to inclusion criteria was strictly maintained, and electronic questionnaires were used to monitor response quality in real time. On‐site guidance was provided to assist patients in completing the surveys, ensuring the authenticity, completeness, and accuracy of the data. In the statistical analysis phase, appropriate analytical methods were applied to process the data, thereby ensuring the reliability of the study findings.

### 2.5. Statistical Analysis

Data analysis was performed using R software (version 4.4.3), primarily utilizing packages including qgraph, NetworkComparisonTest, and bootnet for network analysis. First, descriptive statistics were computed for all variables: continuous variables were presented as mean ± standard deviation, and general demographic data, all categorized, were summarized using frequencies and percentages. Group comparisons of demographic characteristics were conducted using chi‐square tests, with results reported as frequencies and percentages; a *p*‐value of less than 0.05 was considered statistically significant. To evaluate the discriminative power of the scale items, the standard deviation was calculated as a measure of variability, and inter‐item correlations were examined to assess potential redundancy, thereby ensuring the good independence of each item.

#### 2.5.1. Network Structure Estimation

This study employed the qgraph package in R to construct a network structure, utilizing LASSO regularization to analyze the relationships among social anxiety, avoidance, and spiritual well‐being in breast cancer patients, thereby reducing noise and highlighting core connections between key items [28]. The model balanced fit and sparsity through the EBIC criterion (*γ* = 0.5). For network visualization, the Fruchterman–Reingold algorithm was applied, positioning nodes with high centrality near the center and closely related nodes adjacent to one another.

The network comprised 14 nodes, combining two levels of measurement granularity across constructs: two aggregated nodes (total scores of SAS_1 and SAS_2) and 12 item‐level nodes (the 12 items of FACIT‐Sp). This combined granularity was based on three considerations. First, FACIT‐Sp is multidimensional, with three conceptually distinct subdimensions and pre‐analysis item‐level correlations all below 0.25, supporting item‐level analysis for identifying specific intervention targets [27]. Second, both SAS_1 and SAS_2 are unidimensional instruments with strong internal consistency (Cronbach’s *α* > 0.82), and the limited number of items (six for SAS_1) produced unstable estimates when modeled individually. Third, recent methodological work supports that multi‐item indicators (as used for FACIT‐Sp) lead to greater consistency in network properties [29]. The use of item‐level nodes for multidimensional scales has been applied in prior network studies [30].

Edges represented partial correlation coefficients after controlling for other variables, with thickness indicating the strength of associations and color (green for positive and red for negative) indicating direction. The NetworkComparisonTest package was used to compare network structural differences between patients with different surgical approaches, aiming to reveal multidimensional interaction mechanisms among the variables.

#### 2.5.2. Network Characterization

Network characteristics were characterized using three metrics: node strength, bridge strength, and node predictability. Node strength was calculated as the sum of the absolute weights of all edges connected to a given node, reflecting the overall influence of that item within the network. Bridge strength measured the sum of edge weights connecting a node to nodes in other dimensions, serving to evaluate interactions between different dimensions. Node predictability quantified the extent to which a specific item could be predicted by the remaining nodes in the network [31, 32]. Together, these metrics reveal the complex interactive mechanisms among social anxiety, avoidance behavior, and spiritual well‐being in breast cancer patients.

#### 2.5.3. Network Validation

To ensure the reliability and robustness of the network analysis results, this study employed multiple validation methods for comprehensive evaluation [33]. First, edge‐accuracy analysis was conducted to examine the reliability of the network structure. Based on 1000 bootstrap samples, 95% nonparametric confidence intervals (CIs) were calculated for each edge weight. Narrower intervals indicated a higher estimation precision. Second, centrality stability analysis was performed to evaluate the stability of network metrics. The correlation stability coefficient (CS coefficient) was used to quantify the reproducibility of node strength: a CS value >0.25 indicated acceptable stability, while a value >0.5 represented ideal stability. Finally, bootstrap difference tests were applied to compare the differences in edge weights and node strength. If the 95% CI did not include zero, the difference was considered statistically significant (*p*  < 0.05). Together, these methods ensured the scientific validity and reliability of the network analysis results.

## 3. Results

### 3.1. Sample Characteristics and Demographic Analysis

This study distributed 303 paper questionnaires, all of which were returned (100% response rate). After excluding one invalid response, 302 female breast cancer patients were included in the analysis, yielding an effective response rate of 99.67%. As shown in Supporting Information 1 Table S1, most patients (60.9%) were aged 50 or younger, and the majority (91.4%) had been diagnosed within 1 year. Tumor stages were predominantly stage II (47.7%) and stage I (37.7%). A total of 87.1% of patients received postoperative chemotherapy, and surgical approaches were similarly distributed between breast‐conserving/reconstruction (BCS/BR) surgery and mastectomy (50.7% vs. 49.3%, respectively). Scale analysis (Supporting Information 2 Table S2) indicated that all 14 items from the SAS_1, SAS_2, and FACIT‐Sp exhibited reasonable standard deviations. Inter‐item correlations were below 0.25, demonstrating good discriminability and independence among items.

### 3.2. Characteristics and Interconnections of the Social Anxiety, Avoidance, and Spiritual Health Network in Breast Cancer Patients

#### 3.2.1. Network Structure Characteristics

**Figure 1:**
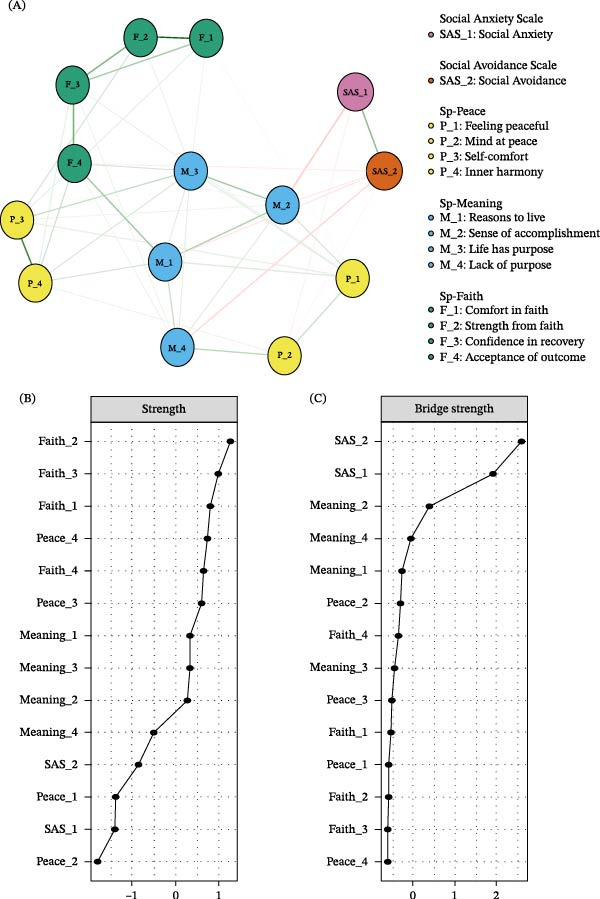
Network diagram of social anxiety, avoidance behaviors, and spiritual health among breast cancer patients (*N* = 302). *Note:* (A) the network structure comprising 14 nodes, constructed using the qgraph package based on a partial correlation matrix. (B) Strength centrality for the 14 nodes in the network. (C) Bridge strength centrality for the 14 nodes in the network.

Figure 1A illustrates the network structure of social anxiety (SAS_1), social avoidance (SAS_2), and spiritual health (FACIT‐Sp) among 302 breast cancer patients. The network comprises 14 nodes, with spiritual health encompassing 12 items across three dimensions: peace, meaning, and faith. The network contains 91 edges (66.7% of possible connections), with an average edge weight of 0.051, indicating relatively dense connectivity but generally weak association strengths between nodes.

#### 3.2.2. Strength Centrality Analysis

Strength centrality results (Figure 1B and Supporting Information 3 Table S3) indicated that Faith_2 (“Strength from faith,” Strength = 1.256) under the Faith dimension was the most influential node, followed by Faith_3 (“Confidence in recovery,” Strength = 0.984) and Faith_1 (“Comfort in faith,” Strength = 0.802).

#### 3.2.3. Bridge Strength Analysis

Bridge strength analysis (Figure 1C and Supporting Information 3 Table S3) revealed that SAS_2 (“Social Avoidance,” Bridge Strength = 2.582) from the SAS_2 dimension had the strongest bridging function, followed by SAS_1 (“Social Anxiety,” Bridge Strength = 1.919) from the Social Anxiety Scale dimension and Meaning_2 (“Sense of accomplishment,” Bridge Strength = 0.382) from the Meaning dimension.

#### 3.2.4. Node Predictability

**Figure 2:**
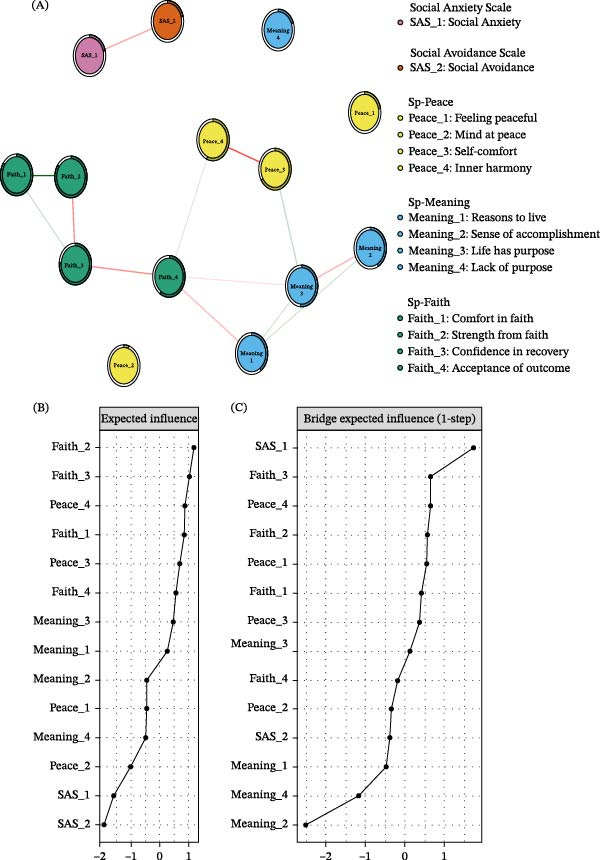
The network structure of social anxiety, avoidance behaviors, and spiritual health among breast cancer patients, with node predictability and influence estimates (*N* = 302). *Note*: (A) network structure with the 14 nodes interactions and attributes. (B) Expected Influence of each node. (C) Bridge Expected Influence.

Node predictability analysis showed that the predictability values of the 14 nodes ranged from 0.044 to 0.850 (Figure 2). Faith_2 (“Strength from faith,” *R* ^2^ = 0.850) under the Faith dimension was the most predictable node, with 85% of its variance explained by other items in the network. This was followed by Faith_1 (“Comfort in faith,” *R* ^2^ = 0.849) and Faith_3 (“Confidence in recovery,” *R* ^2^ = 0.759) (Supporting Information 3 Table S3).

#### 3.2.5. Analysis of Network Characteristic Differences by Surgical Approach Level

**Figure 3:**
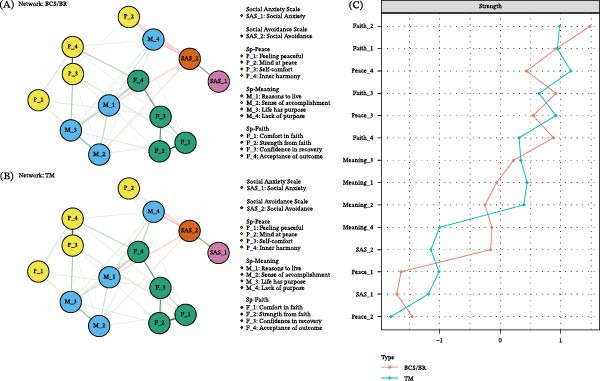
Comparison of network structure and influence by surgical approach level. *Note*: BCS, breast‐conserving surgery, BR, breast reconstruction; TM, total mastectomy. (A) Network for BCS/BR (*N* = 153), (B) network for TM (*N* = 149), and (C) strength by surgical approach level.

Network comparison between the BCS/BR and total mastectomy (TM) groups revealed no significant differences in global network structure (*M* = 0.225, *p*  = 0.714) or overall connectivity strength (5.54 vs. 5.69, *S* = 0.148, *p*  = 0.645) (Figure 3).

However, edge invariance testing identified nine edges with significant between‐group differences (*p*  < 0.05; Supporting Information 4 Table S4), primarily involving relationships between social avoidance (SAS_2) and peace of mind (e.g., Peace_2 and Peace_3), as well as between inner harmony (Peace_4) and meaning in life (e.g., Meaning_1 and Meaning_3).

**Figure 4:**
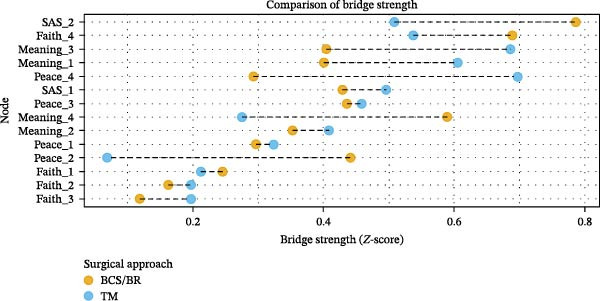
Comparison of bridge strength by surgical approach level. *Note*: BCS, breast‐conserving surgery, BR, breast reconstruction; TM, total mastectomy.

Centrality analysis indicated that except for Peace_4 (inner harmony), which showed significantly higher strength centrality in the TM group (*p*  = 0.030), no other nodes differed significantly between groups. Although the mean bridge strength did not differ overall (*t* = −0.033, *p*  = 0.975), distinct patterns emerged at the node level (Figure 4): the TM group exhibited higher bridge strength for Peace_4, Meaning_3, and Meaning_1, whereas the BCS/BR group showed stronger bridge roles for Peace_2, Meaning_4, and SAS_2 (Supporting Information 5 Table S5). Of note, the edge‐level comparisons were not adjusted for multiple testing (e.g., Bonferroni correction); therefore, the nine identified edge differences should be considered exploratory and require validation in larger samples.

### 3.3. Analysis of Network Accuracy and Stability

#### 3.3.1. Edge Weight Accuracy

Bootstrap analysis evaluated the stability of the network estimates. The results showed wide CIs for edge weights (mean 95% CI: [−0.0075, 0.0514]), with 84.6% of intervals including zero, suggesting instability in most edge estimates. The median edge strength was 0.1258, indicating that the network is primarily composed of weak connections. These findings suggest that the interpretation of specific edges—particularly weak ones—should be made with caution, and future studies with larger samples are needed for more precise estimates.

#### 3.3.2. Centrality Stability

**Figure 5:**
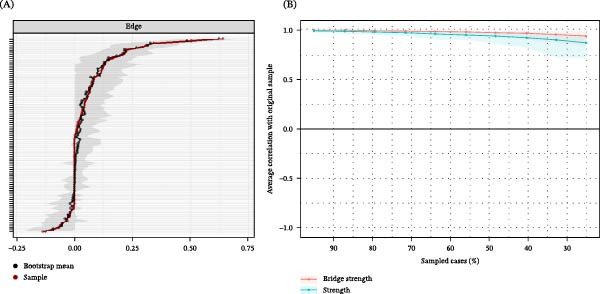
Analysis of social anxiety, avoidance behaviors, and the accuracy and stability of the mental health network (*N* = 302). *Note*: (A) accuracy analysis of the edge weights. (B) Stability analysis of the centrality indice.

The centrality stability test (Figure 5B) showed that both strength and bridge strength centralities remained stable (CS coefficient = 0.748 > 0.5). This supports the reliability of key nodes such as Faith_2 (strength = 1.256) and SAS_2 (Bridge Strength = 2.582) in occupying central roles within the network.

### 3.4. Tests for Differences in Network Characteristics

This study employed a bootstrap resampling method (nBoots = 1000) to examine differences in edge weights, node strength centrality, and bridge strength centrality within the network. Results are shown in Supporting Information 6 Figure S1, Supporting Information 7 Figure S2, and Supporting Information 8 Figure S3.

#### 3.4.1. Edge Weight Differences

Supporting Information 6 Figure S1 presents the differential test results for edge weights. In the matrix plot, black squares indicate statistically significant differences between the edges. The strongest and most stable connections were primarily observed within the same psychological construct—for instance, between Faith_1 and Faith_2 (0.640, 95% CI [0.494, 0.769]) within the faith dimension and between Peace_3 and Peace_4 (0.502, 95% CI [0.367, 0.606]) within the peace dimension, indicating strong internal coherence within these core constructs. In contrast, significant cross‐dimensional connections were relatively scarce, with the strongest bridging link observed between Meaning_1 and Faith_4 (0.225, 95% CI [0.102, 0.321]). Additionally, one negative edge was observed between SAS_1 and Meaning_2 (−0.136, 95% CI [−0.226, −0.023]). Given the wide CIs and the overall instability of edge estimates (Section 3.3.1), this finding should be interpreted with caution and requires replication.

#### 3.4.2. Node Strength Centrality Differences

As shown in Supporting Information 7 Figure S2, the diagonal of the matrix displays the strength values of each node, while gray/black boxes indicate whether the difference between node pairs is significant. The results demonstrate that the strength centrality of Faith_2 (Strength = 1.256) and Faith_3 (Strength = 0.984) differed significantly from that of most other nodes in the network, further confirming their central roles.

#### 3.4.3. Node Bridge Strength Centrality Differences

Supporting Information 8 Figure S3 illustrates the differences in bridge strength across nodes. The analysis revealed that the social avoidance node SAS_2 (Bridge Strength = 2.582) had significantly higher bridge strength than most other nodes, underscoring its critical role in connecting the different dimensions of social anxiety/avoidance and spiritual well‐being.

## 4. Discussion

This study employed network analysis to elucidate the complex interactive system involving social anxiety, avoidance behaviors, and spiritual well‐being among breast cancer patients. The main findings are as follows:

1. The spiritual well‐being exhibited the highest strength centrality among all nodes in the network. Specifically, the faith‐related items “Strength from faith” (Faith_2) and “Confidence in recovery” (Faith_3) showed the greatest strength centrality. This indicates that these spiritual well‐being items are highly interconnected with other psychological variables in the network, aligning with existing studies that spiritual resources play a supportive role in cancer adaptation [34, 35]. However, because the network combined item‐level nodes for spiritual well‐being and aggregated nodes for social anxiety and avoidance, direct comparisons of centrality estimates across these constructs should be interpreted with caution. Nevertheless, the pattern suggests that spiritual well‐being—particularly the faith dimension—occupies a central position in the psychological adaptation network.
2. Social avoidance behavior (SAS_2) demonstrated the strongest bridge centrality among all nodes. This suggests a potentially important role for social avoidance in connecting spiritual well‐being with symptoms of social anxiety, extending previous research that focused primarily on the independent effects of symptoms. Social avoidance may function not only as a behavioral manifestation but also as a potential mediator between spiritual resources and emotional states [12, 36]. Importantly, despite the asymmetric node granularity across constructs, the substantially higher bridge strength of SAS_2 (2.582) relative to the next highest value (SAS_1 = 1.919) supports the robustness of this finding.
3. Although different surgical approaches (BCS/BR vs. TM) did not alter the overall network structure, they shaped distinct psychological adaptation pathways, as reflected by between‐group differences in node‐level bridge strength. This observation resonates with studies reporting that surgery type influences psychological outcomes [37], while our study illustrates the underlying mechanism from a network perspective. Nevertheless, given the modest subgroup sizes (*n* = 153 and *n* = 149) and the lack of multiple testing correction, these subgroup differences should be viewed as exploratory and warrant replication in larger samples.
4. The network was primarily composed of weak connections, and edge estimation precision was limited: 84.6% of edge CIs contained zero, indicating low precision for many individual edges. This pattern has been observed in other network studies with moderate sample sizes. However, node centrality indices (strength and bridge strength) showed good stability (CS = 0.748), supporting the strategy of focusing interpretive efforts on node‐level rather than edge‐level characteristics. This finding is consistent with discussions in network psychometrics regarding the “ubiquity of weak connections” while also highlighting that core nodes may still offer reliable interpretive value even when the global network is unstable [30].

In summary, this research provides a systems perspective on the interactions among key psychological variables in breast cancer patients. It not only supplements empirical evidence regarding the pathways linking spiritual health and social behavior but also offers a theoretical basis for developing precise psychological intervention strategies.

## 5. Implications

The findings of this study offer important implications for clinical practice, future research, and health education, as outlined below.

### 5.1. Clinical Practice

1. Integrate spiritual health into routine psychological assessment, with emphasis on core dimensions such as “strength from faith” and “confidence in recovery.” Healthcare providers may use tools like the FACIT‐Sp or open‐ended questions (e.g., “what gives you strength to face the illness?”) to promptly identify patients’ spiritual resources and deliver targeted interventions such as mindfulness‐based spiritual training or meaning‐centered therapy [38, 39].
2. Prioritize intervention on social avoidance, a key bridge between spiritual health and emotional symptoms. Techniques such as behavioral activation (e.g., graded exposure) and cognitive restructuring may be employed to disrupt the avoidance cycle and facilitate the translation of spiritual resources into positive emotional regulation.
3. Develop surgery‐specific support strategies: patients undergoing TM may benefit from exploring existential issues (e.g., meaning in life and inner harmony), whereas those receiving BCS/BR surgery may require enhanced emotion regulation and social skills training for tailored psychological support [40].

### 5.2. Research Directions

Future studies should validate the network structure in larger samples and incorporate biological markers (e.g., cortisol levels) and socioenvironmental factors to develop causal network models. In addition, randomized controlled trials targeting central nodes (e.g., Faith_2 and SAS_2) are needed to compare the efficacy of different intervention strategies (e.g., spiritual reinforcement vs. behavioral activation) in modulating overall network connectivity.

### 5.3. Health Education

Health education programs for breast cancer patients should incorporate spiritual health and social behavior management. Key protective factors identified via network analysis can be disseminated through peer support groups or digital platforms (e.g., mHealth apps) to enhance self‐management competence. Meanwhile, training medical staff in network thinking may help identify “leverage points” in psychological systems and improve the efficiency of resource allocation.

### 5.4. Limitations

This study has several limitations:

1. Limited sample representativeness: the sample was recruited from a single tertiary hospital in Sichuan Province, with a majority of Han Chinese and married patients. This may limit the generalizability of the findings to populations from other regions, cultures, or social backgrounds. Given that faith and spiritual well‐being vary substantially across cultures, the limited generalizability to non‐Han or non‐Chinese populations should be particularly emphasized. Future studies should adopt multi‐center sampling to enhance external validity.
2. Constraints of cross‐sectional design: the network analysis was based on cross‐sectional data, which precludes causal inferences or examination of dynamic changes among variables. Longitudinal or experimental studies are needed to complement these findings. Moreover, the subgroup network comparisons (BCS/BR vs. TM) were based on modest sample sizes (*n* = 153 and *n* = 149), and the edge‐level differences were not corrected for multiple testing; thus, these findings should be interpreted as exploratory.
3. Reliance on self‐report measures: self‐report scales may introduce social desirability or recall biases; incorporating objective or interview data could improve reliability. Additionally, the modified 5‑point version of the SAS_2 lacked formal validation beyond internal consistency, limiting comparability with dichotomous‑based studies. Finally, SAS_1 (1975) and SAS_2 (1969) were designed for general populations; although validated Chinese adaptations were used, they may not fully capture illness‑specific or body image‑related social avoidance in contemporary breast cancer patients. Future research should develop condition‑specific measures.
4. Precision of network estimates: Edge weight CIs were wide (mean 95% CI: [−0.0075, 0.0514]), and 84.6% of connections included zero, indicating instability in the estimation of many weak edges. This reflects the complexity of psychological systems but underscores the need for larger samples to improve estimation robustness. Additionally, the network adopted asymmetric measurement granularity across constructs: item‐level nodes for spiritual well‐being versus aggregated nodes for social anxiety and avoidance. Although this choice was justified in the Methods section based on the multidimensional structure of FACIT‐Sp and the unidimensionality of the other scales, it may limit direct comparisons of centrality estimates across constructs. Future research should consider consistent item‐level or dimension‐level modeling across all constructs to assess the robustness of our findings.

## 6. Conclusion

This study employed network analysis to systematically elucidate the interactive mechanisms among social anxiety, avoidance behaviors, and spiritual well‐being in breast cancer patients. Key findings indicate that spiritual well‐being—particularly the faith dimension—occupies a central position in the psychological adaptation network, while social avoidance demonstrates the strongest bridge centrality linking symptoms and resources. Although the surgical approach did not alter the overall network structure, it modulated interaction pathways between nodes, underscoring the need for personalized interventions. Despite limitations in sample representativeness and estimation precision, the high stability of centrality indices supports the reliability of key intervention targets. Notwithstanding the methodological limitations discussed above, the high stability of centrality indices supports the reliability of key intervention targets. Future research should focus on large‐sample validation, exploration of causal mechanisms, and interventions targeting network nodes to advance precision‐ and systems‐oriented psychological support for breast cancer patients.

## Funding

The authors declare that this research received no specific grant from any funding agency in the public, commercial, or not‐for‐profit sectors.

## Ethics Statement

This study was conducted as an anonymous investigation, adhering to ethical guidelines without involving any unethical behavior or human clinical trials. The Biomedical Ethics Committee of West China Hospital, Sichuan University, thoroughly reviewed and granted approval for this study (Approval Number 2023(213)).

## Conflicts of Interest

The authors declare no conflicts of interest.

## Supporting Information

Additional supporting information can be found online in the Supporting Information section.

## Supporting information


**Supporting Information 1** Table S1: Characteristics of breast cancer patients (*N* = 302).

**Supporting Information 2** Table S2: Social Anxiety Scale (SAS_1), Social Avoidance Scale (SAS_2), functional assessment of chronic illness therapy‐spiritual well‐being (FACIT‐Sp), and descriptive statistics for each item in breast cancer patients.

**Supporting Information 3** Table S3: Network of social anxiety, avoidance, and spiritual well‐being in breast cancer patients: nodal list with centrality and predictability estimates.

**Supporting Information 4** Table S4: Edge weight difference test between surgical approach level networks.

**Supporting Information 5** Table S5: Comparison of bridge strength differences by surgical approach among the 14 nodes.

**Supporting Information 6** Figure S1: Bootstrapped difference test for edge weights. *Note:* the axes correspond to individual edges within the network of social anxiety, avoidance behavior, and spiritual well‐being. Gray cells indicate nonsignificant differences, whereas black cells indicate significant differences.

**Supporting Information 7** Figure S2: Bootstrapped difference test for node strength centrality. *Note:* the *x* and *y* axes correspond to individual nodes within the network of social anxiety, avoidance behavior, and spiritual well‐being. The diagonal displays the strength centrality values for each node. Nonsignificant and significant differences are indicated by gray and black cells, respectively.

**Supporting Information 8** Figure S3: Bootstrapped difference test for node bridge strength centrality. *Note:* the axes correspond to individual nodes within the network of social anxiety, avoidance behavior, and spiritual well‐being. The diagonal displays the bridge strength centrality values. Nonsignificant and significant differences are indicated by gray and black cells, respectively.

## Data Availability

The data that support the findings of this study are available upon request from the corresponding author. The data are not publicly available due to privacy or ethical restrictions.
