# Supplementary material for: Network Analysis of Social Anxiety, Avoidant Behavior, and Spiritual Well‐Being in Breast Cancer Patients: A Pathway to Targeted Psychosocial Interventions
Source: Depress Anxiety. 2026 Aug 7;2026:9076098. doi: 10.1155/da/9076098 (PMC13448774; doi:10.1155/da/9076098)
Supplement: Supplementary file 1 [file DA-2026-9076098-s002.pdf]

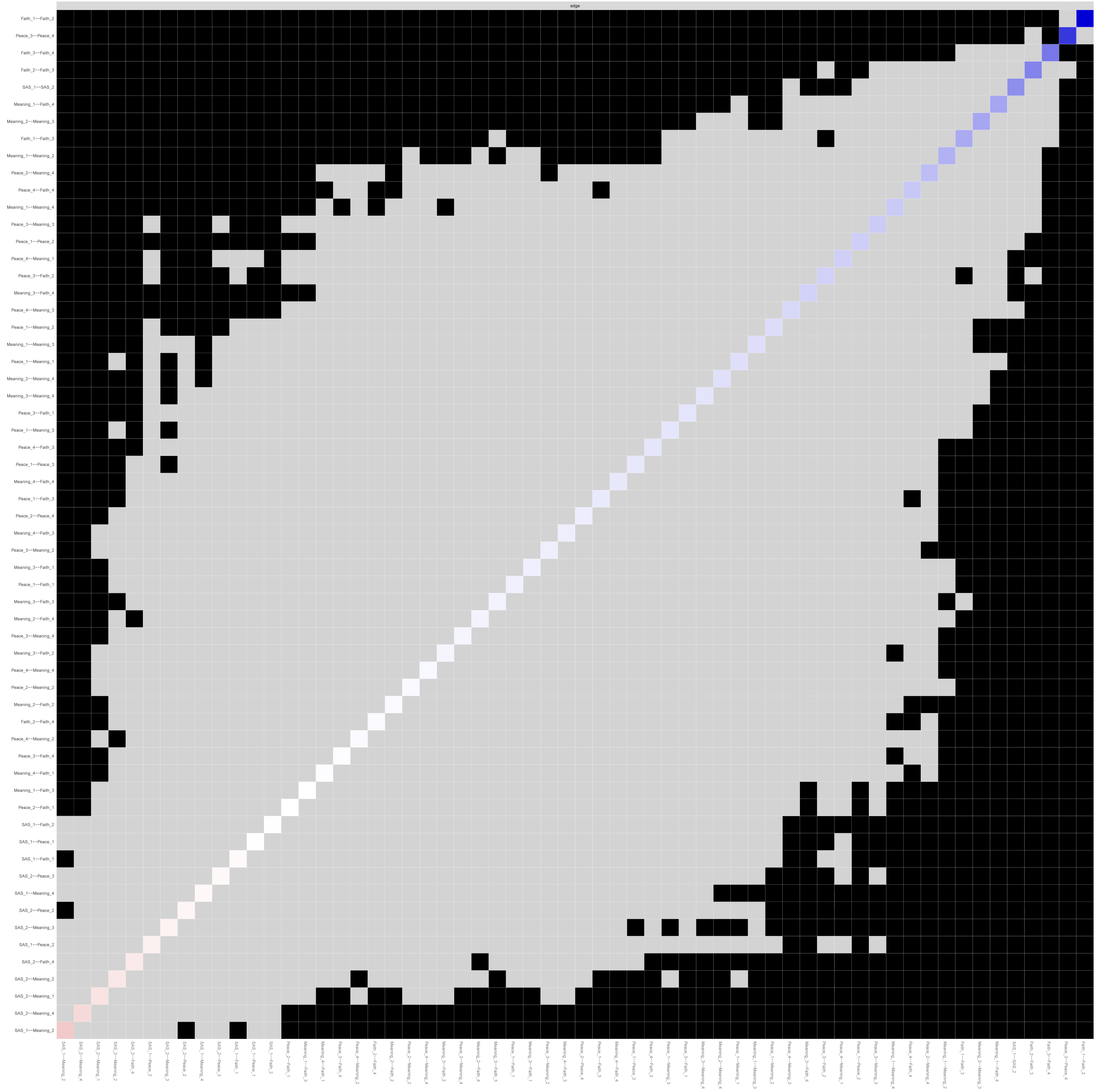
edge
Faith_1--Faith_2
Peace_3--Peace_4
Faith_3--Faith_4
Faith_2--Faith_3
SAS_1--SAS_2
Meaning_1--Faith_4
Meaning_2--Meaning_3
Faith_1--Faith_3
Meaning_1--Meaning_2
Peace_2--Meaning_4
Peace_4--Faith_4
Meaning_1--Meaning_4
Peace_3--Meaning_3
Peace_1--Peace_2
Peace_4--Meaning_1
Peace_3--Faith_2
Meaning_3--Faith_4
Peace_4--Meaning_3
Peace_1--Meaning_2
Meaning_1--Meaning_3
Peace_1--Meaning_1
Meaning_2--Meaning_4
Meaning_3--Meaning_4
Peace_3--Faith_1
Peace_1--Meaning_3
Peace_4--Faith_3
Peace_1--Peace_3
Meaning_4--Faith_4
Peace_1--Faith_3
Peace_2--Peace_4
Meaning_4--Faith_3
Peace_3--Meaning_2
Meaning_3--Faith_1
Peace_1--Faith_1
Meaning_3--Faith_3
Meaning_2--Faith_4
Peace_3--Meaning_4
Meaning_3--Faith_2
Peace_4--Meaning_4
Peace_2--Meaning_2
Meaning_2--Faith_2
Faith_2--Faith_4
Peace_4--Meaning_2
Peace_3--Faith_4
Meaning_4--Faith_1
Meaning_1--Faith_3
Peace_2--Faith_1
SAS_1--Faith_2
SAS_1--Peace_1
SAS_1--Faith_1
SAS_2--Peace_3
SAS_1--Meaning_4
SAS_2--Peace_2
SAS_2--Meaning_3
SAS_1--Peace_2
SAS_2--Faith_4
SAS_2--Meaning_2
SAS_2--Meaning_1
SAS_2--Meaning_4
SAS_1--Meaning_2
