# Supplementary material for: Network Analysis of Social Anxiety, Avoidant Behavior, and Spiritual Well‐Being in Breast Cancer Patients: A Pathway to Targeted Psychosocial Interventions
Source: Depress Anxiety. 2026 Aug 7;2026:9076098. doi: 10.1155/da/9076098 (PMC13448774; doi:10.1155/da/9076098)
Supplement: Supplementary file 2 [file DA-2026-9076098-s003.pdf]

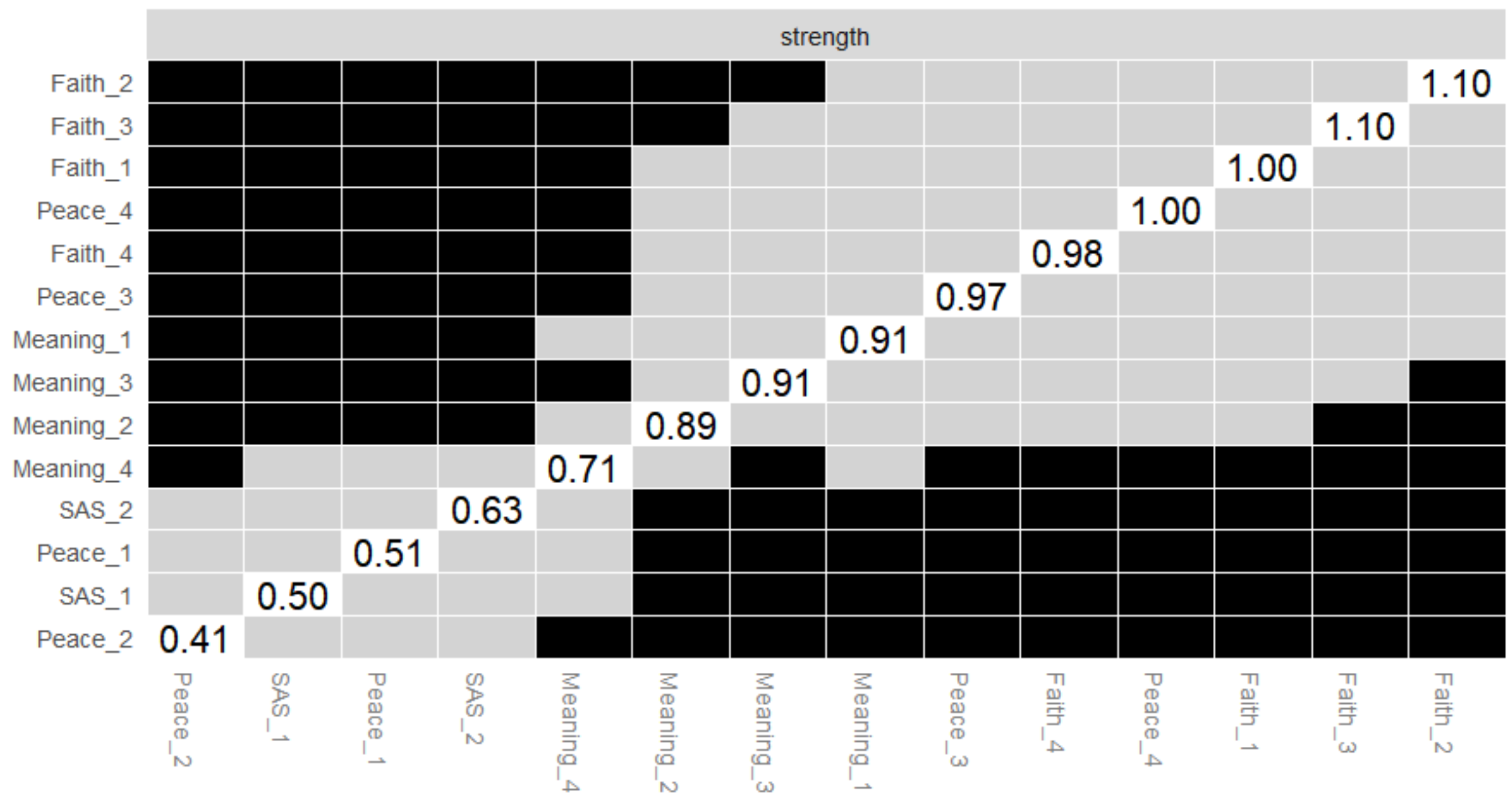
strength
Faith_2
Faith_3
Faith_1
Peace_4
Faith_4
Peace_3
Meaning_1
Meaning_3
Meaning_2
Meaning_4
SAS_2
Peace_1
SAS_1
Peace_2
1.10
1.10
1.00
1.00
0.98
0.97
0.91
0.91
0.89
0.71
0.63
0.51
0.50
0.41
Peace_2
SAS_1
Peace_1
SAS_2
Meaning_4
Meaning_2
Meaning_3
Meaning_1
Peace_3
Faith_4
Peace_4
Faith_1
Faith_3
Faith_2
