# Supplementary material for: Network Analysis of Social Anxiety, Avoidant Behavior, and Spiritual Well‐Being in Breast Cancer Patients: A Pathway to Targeted Psychosocial Interventions
Source: Depress Anxiety. 2026 Aug 7;2026:9076098. doi: 10.1155/da/9076098 (PMC13448774; doi:10.1155/da/9076098)
Supplement: Supplementary file 3 [file DA-2026-9076098-s004.pdf]

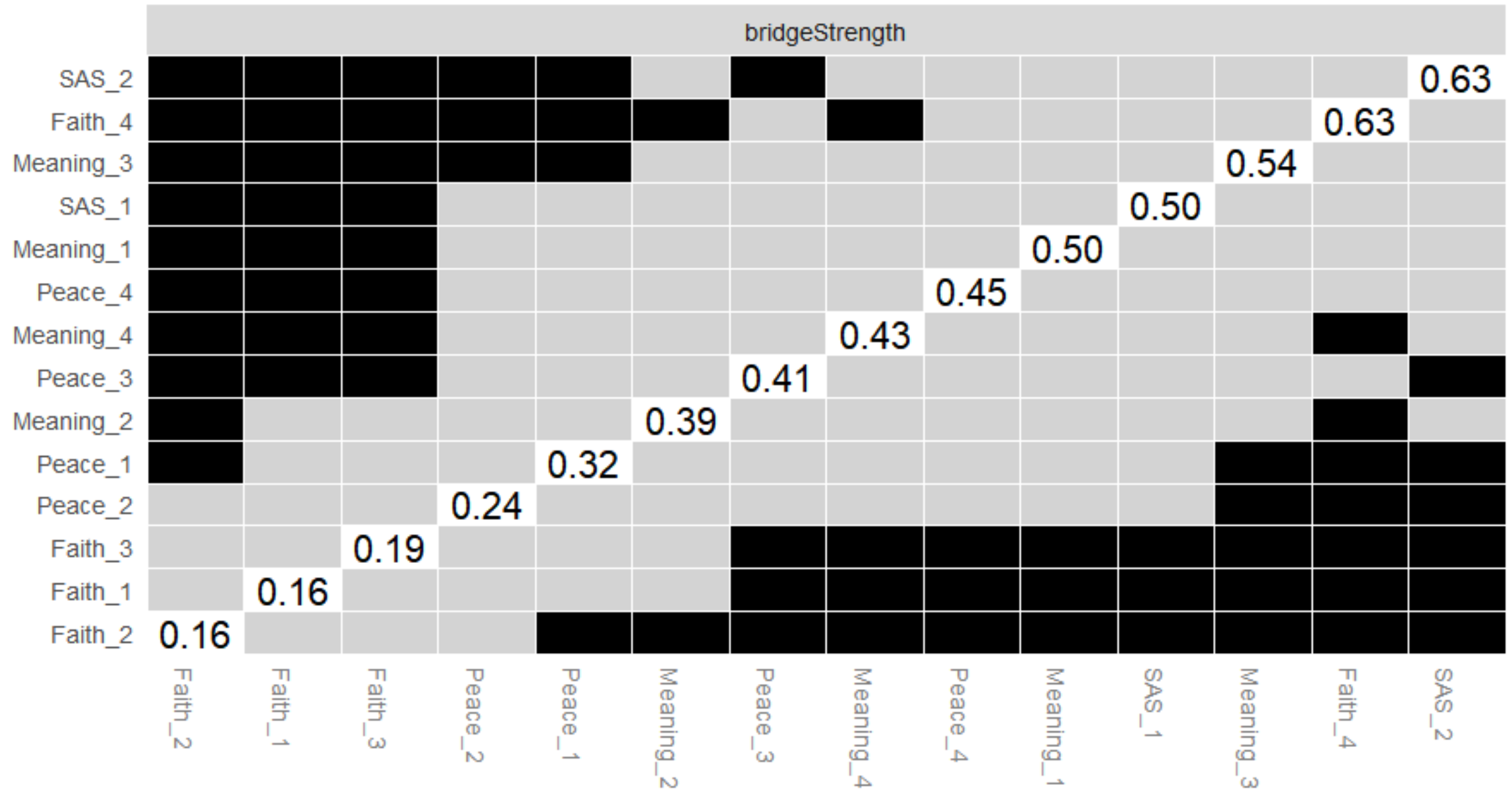
bridgeStrength
SAS_2
Faith_4
Meaning_3
SAS_1
Meaning_1
Peace_4
Meaning_4
Peace_3
Meaning_2
Peace_1
Peace_2
Faith_3
Faith_1
Faith_2
0.63
0.63
0.54
0.50
0.50
0.45
0.43
0.41
0.39
0.32
0.24
0.19
0.16
0.16
Faith_2
Faith_1
Faith_3
Peace_2
Peace_1
Meaning_2
Peace_3
Meaning_4
Peace_4
Meaning_1
SAS_1
Meaning_3
Faith_4
SAS_2
