# Supplementary material for: Network Analysis of Social Anxiety, Avoidant Behavior, and Spiritual Well‐Being in Breast Cancer Patients: A Pathway to Targeted Psychosocial Interventions
Source: Depress Anxiety. 2026 Aug 7;2026:9076098. doi: 10.1155/da/9076098 (PMC13448774; doi:10.1155/da/9076098)
Supplement: Supplementary file 4 [file DA-2026-9076098-s001.docx]

**Table 5. Comparison of Bridge Strength Differences by Surgical Approach among the 14 Nodes**

| **Node** | **BCS/BR** | **TM** | **Difference** |
| --- | --- | --- | --- |
| SAS_1 | 0.429 | 0.497 | -0.068 |
| SAS_2 | 0.786 | 0.508 | 0.278 |
| Peace_1 | 0.297 | 0.325 | -0.028 |
| Peace_2 | 0.441 | 0.070 | 0.371 |
| Peace_3 | 0.436 | 0.458 | -0.022 |
| Peace_4 | 0.293 | 0.697 | -0.404 |
| Meaning_1 | 0.401 | 0.606 | -0.205 |
| Meaning_2 | 0.353 | 0.409 | -0.056 |
| Meaning_3 | 0.405 | 0.686 | -0.281 |
| Meaning_4 | 0.589 | 0.276 | 0.314 |
| Faith_1 | 0.246 | 0.212 | 0.034 |
| Faith_2 | 0.163 | 0.197 | -0.034 |
| Faith_3 | 0.119 | 0.197 | -0.078 |
| Faith_4 | 0.689 | 0.538 | 0.151 |

***Note：***BCS: Breast-conserving surgery, BR: Breast reconstruction; TM: Total mastectomy.
