# Supplementary material for: Network Analysis of Social Anxiety, Avoidant Behavior, and Spiritual Well‐Being in Breast Cancer Patients: A Pathway to Targeted Psychosocial Interventions
Source: Depress Anxiety. 2026 Aug 7;2026:9076098. doi: 10.1155/da/9076098 (PMC13448774; doi:10.1155/da/9076098)
Supplement: Supplementary file 5 [file DA-2026-9076098-s005.docx]

Table 1. Characteristics of Breast Cancer Patients (*N* = 302)

| **Characteristics** | **Frequency(*N*)** | **Percentage(%)** |
| --- | --- | --- |
| **Age (years)** |  |  |
| ≤50 | 184 | 60.9 |
| ＞50 | 118 | 39.1 |
| **Ethnicity** |  |  |
| Han | 286 | 94.7 |
| Others | 16 | 5.3 |
| **Marital status*** |  |  |
| Married | 281 | 93.0 |
| Single | 21 | 7.0 |
| **Educational level** |  |  |
| Bachelor's Degree and Above | 88 | 29.1 |
| junior college | 52 | 17.2 |
| Secondary Education and Below | 162 | 53.6 |
| **Religious beliefs**** |  |  |
| No | 278 | 92.1 |
| Yes | 24 | 7.9 |
| **Current work status** |  |  |
| Retired | 106 | 35.1 |
| Employed | 105 | 34.8 |
| Unemployed | 91 | 30.1 |
| **Family residence location** |  |  |
| Urban | 213 | 70.5 |
| Rural | 51 | 16.9 |
| Town | 38 | 12.6 |
| **Residential status** |  |  |
| Living with family or friends | 291 | 96.4 |
| Living alone | 11 | 3.6 |
| **Level of family care and support since the illness onset** | | |
| Very much | 234 | 77.5 |
| Quite a lot | 56 | 18.5 |
| Relatively little | 12 | 4.0 |
| **Personality types** |  |  |
| Ambivert type | 134 | 44.4 |
| Extroversion tendency type | 84 | 27.8 |
| Introversion tendency type | 41 | 13.6 |
| Typical extrovert type | 36 | 11.9 |
| Typical introvert type | 7 | 2.3 |
| **Per capita monthly household income** |  |  |
| 5000 yuan and below | 117 | 38.7 |
| 5001-8000 yuan | 73 | 24.2 |
| 8001 yuan and above | 112 | 37.1 |
| **Disease duration (years)** |  |  |
| ＜1 | 276 | 91.4 |
| ≥1 | 26 | 8.6 |
| **Tumor stage** |  |  |
| Stage I | 114 | 37.7 |
| Stage II | 144 | 47.7 |
| Stage III | 44 | 14.6 |
| **Sequence of surgery and chemotherapy** |  |  |
| Primary debulking surgery (PDS) followed by chemotherapy | 263 | 87.1 |
| Neoadjuvant chemotherapy (NAC) followed by surgery | 39 | 12.9 |
| **Breast surgery approaches** |  |  |
| Breast-conserving/Breast reconstruction | 153 | 50.7 |
| Mastectomy | 149 | 49.3 |
| **Axillary surgery approaches** |  |  |
| Lymph node dissection | 118 | 39.1 |
| Sentinel lymph node biopsy | 184 | 60.9 |
| **The total number of chemotherapy cycles required to be received** | | |
| 8 | 91 | 30.1 |
| 6 | 83 | 27.5 |
| 4 | 126 | 41.7 |
| Other | 2 | 0.7 |
| **Total number of chemotherapy sessions (including this one)** | | |
| ＜5 | 140 | 46.4 |
| ≥5 | 162 | 53.6 |
| Note. *Single indicated separated, divorced, widowed, or never married, and married indicated married or partnered.  **“Yes” indicates having a religious belief. Among the 24 participants who reported a religious belief, 22 specified Buddhism and 2 specified Taoism. The FACIT-Sp Faith subscale measures spiritual well‑being broadly, not limited to organized religion. | | |
