# Supplementary material for: Network Analysis of Social Anxiety, Avoidant Behavior, and Spiritual Well‐Being in Breast Cancer Patients: A Pathway to Targeted Psychosocial Interventions
Source: Depress Anxiety. 2026 Aug 7;2026:9076098. doi: 10.1155/da/9076098 (PMC13448774; doi:10.1155/da/9076098)
Supplement: Supplementary file 6 [file DA-2026-9076098-s006.docx]

Table 2. Social Anxiety Scale (SAS1), Social Avoidance Scale (SAS2), Functional Assessment of Chronic Illness Therapy-Spiritual Well-Being (FACIT-Sp), and Descriptive Statistics for Each Item in Breast Cancer Patients

| **Items** |  | **Mean** | **SD** |
| --- | --- | --- | --- |
| **Social Anxiety Scale (SAS_1_)** | |  |  |
| Social Anxiety | | 9.37 | 4.88 |
| SAS_1__1 | I need time to overcome my shyness/timidity in a new environment | 1.80 | 1.16 |
| SAS_1__2 | I usually mess things up when someone is watching me | 1.31 | 1.08 |
| SAS_1__3 | I become embarrassed very easily | 1.48 | 1.097 |
| SAS_1__4* | I find it easy to talk to strangers | 1.58 | 1.08 |
| SAS_1__5 | I feel nervous when speaking in front of a crowd | 1.65 | 1.12 |
| SAS_1__6 | Being surrounded by a large group of people makes me nervous | 1.55 | 1.09 |
| **Social Avoidance Scale (SAS_2_)** | | | |
| Social Avoidance | | 24.82 | 8.40 |
| SAS_2__1 | I try to attend as few social events as possible | 2.31 | 1.20 |
| SAS_2__2* | I don’t particularly try to avoid people | 1.74 | 1.13 |
| SAS_2__3 | I try to avoid talking to others unless they are close acquaintances | 1.55 | 1.22 |
| SAS_2__4* | I take the opportunity to meet new people whenever possible | 1.99 | 1.06 |
| SAS_2__5 | I often wish to be alone and away from others | 1.45 | 1.11 |
| SAS_2__6* | Even if a room is full of strangers, I would probably still go in | 1.91 | 1.07 |
| SAS_2__7 | I try to stay away from crowded places | 2.37 | 1.14 |
| SAS_2__8* | I am glad to talk with my leader or elder if they wish to speak with me | 1.30 | 0.92 |
| SAS_2__9 | I like to stay away from crowds | 1.57 | 1.08 |
| SAS_2__10* | Talking with people at social gatherings is not a problem for me | 1.51 | 1.04 |
| SAS_2__11 | I often find excuses to avoid social activities | 1.75 | 1.13 |
| SAS_2__12* | I like to introduce people to each other | 1.94 | 1.06 |
| SAS_2__13 | I try to avoid formal social occasions | 1.85 | 1.05 |
| SAS_2__14* | I usually attend every social activity I can | 1.57 | 1.02 |
| **Spiritual well being(Sp)** | | | |
| Peace | | 11.17 | 2.58 |
| Peace_1 | I feel peaceful | 2.43 | 0.94 |
| Peace_2* | My mind is not at peace | 3.05 | 0.82 |
| Peace_3 | I can comfort myself from within | 2.8 | 0.95 |
| Peace_4 | I am able to live in harmony with myself | 2.89 | 0.96 |
| Meaning | | 11.16 | 2.93 |
| Meaning_1 | I have reasons to live | 3.2 | 0.87 |
| Meaning_2 | I feel a sense of accomplishment in my life | 2.26 | 1.08 |
| Meaning_3 | I feel that my life has purpose | 2.48 | 1.06 |
| Meaning_4* | My life lacks meaning and purpose | 3.22 | 0.90 |
| Faith | | 11.26 | 3.48 |
| Faith_1 | I find comfort in my faith or spiritual beliefs | 2.67 | 0.97 |
| Faith_2 | I gain strength from my faith or spiritual beliefs | 2.72 | 0.98 |
| Faith_3 | My faith or spiritual beliefs strengthen my confidence in recovery | 2.84 | 0.96 |
| Faith_4 | I know that, no matter how my illness changes, everything will be all right | 3.03 | 0.92 |
| *Note:* SD: standard deviations, *：Reverse-scored item(s) . | | | |
