# Supplementary material for: Network Analysis of Social Anxiety, Avoidant Behavior, and Spiritual Well‐Being in Breast Cancer Patients: A Pathway to Targeted Psychosocial Interventions
Source: Depress Anxiety. 2026 Aug 7;2026:9076098. doi: 10.1155/da/9076098 (PMC13448774; doi:10.1155/da/9076098)
Supplement: Supplementary file 7 [file DA-2026-9076098-s007.docx]

Table 3: Network of Social Anxiety, Avoidance, and Spiritual Well-being in Breast Cancer Patients: Nodal List with Centrality and Predictability Estimates

| **Nodes** | **Variables** | **Strength** | **Bridge strength** | **Expected influence** | **Bridge expected influence (1-step)** | **Predictability (R^2^)** |
| --- | --- | --- | --- | --- | --- | --- |
| SAS_1 | Social Anxiety | -1.414 | 1.919 | -1.592 | 1.727 | 0.211 |
| SAS_2 | Social Avoidance | -0.854 | 2.582 | -1.938 | -0.378 | 0.199 |
| Peace_1 | Feeling peaceful | -1.389 | -0.585 | -0.460 | 0.556 | 0.217 |
| Peace_2 | Mind at peace | -1.808 | -0.306 | -1.011 | -0.332 | 0.044 |
| Peace_3 | Self-comfort | 0.599 | -0.526 | 0.704 | 0.366 | 0.662 |
| Peace_4 | Inner harmony | 0.739 | -0.613 | 0.882 | 0.644 | 0.562 |
| Meaning_1 | Reasons to live | 0.344 | -0.258 | 0.267 | -0.485 | 0.399 |
| Meaning_2 | Sense of accomplishment | 0.266 | 0.382 | -0.449 | -2.518 | 0.423 |
| Meaning_3 | Life has purpose | 0.344 | -0.450 | 0.467 | 0.125 | 0.511 |
| Meaning_4 | Lack of purpose | -0.509 | -0.044 | -0.483 | -1.164 | 0.188 |
| Faith_1 | Comfort in faith | 0.802 | -0.545 | 0.851 | 0.429 | 0.849 |
| Faith_2 | Strength from faith | 1.256 | -0.591 | 1.178 | 0.574 | 0.850 |
| Faith_3 | Confidence in recovery | 0.984 | -0.613 | 1.033 | 0.644 | 0.759 |
| Faith_4 | Acceptance of outcome | 0.642 | -0.352 | 0.549 | -0.186 | 0.592 |
