# Supplementary material for: Network Analysis of Social Anxiety, Avoidant Behavior, and Spiritual Well‐Being in Breast Cancer Patients: A Pathway to Targeted Psychosocial Interventions
Source: Depress Anxiety. 2026 Aug 7;2026:9076098. doi: 10.1155/da/9076098 (PMC13448774; doi:10.1155/da/9076098)
Supplement: Supplementary file 8 [file DA-2026-9076098-s008.docx]

**Table 4. Edge weight difference test between surgical approach level networks.**

| Var1: BCS/BR | Var1: TM | *P*-value | Test statistic E |
| --- | --- | --- | --- |
| SAS_2 | Peace_2 | 0.031 | 0.137 |
| SAS_2 | Peace_3 | 0.035 | 0.089 |
| Peace_2 | Peace_3 | 0.012 | 0.082 |
| Peace_1 | Peace_4 | 0.022 | 0.133 |
| Peace_4 | Meaning_1 | 0.018 | 0.224 |
| SAS_1 | Meaning_2 | 0.035 | 0.218 |
| Peace_4 | Meaning_3 | 0.016 | 0.225 |
| Meaning_4 | Faith_1 | 0.017 | 0.082 |
| Meaning_4 | Faith_2 | 0.036 | 0.046 |

***注：***BCS: Breast-conserving surgery, BR: Breast reconstruction; TM: Total mastectomy.
